# Supplementary material for: wtf genes are prolific dual poison-antidote meiotic drivers
Source: eLife. 2017 Jun 20;6:e26033. doi: 10.7554/eLife.26033 (PMC5478261; doi:10.7554/eLife.26033)
Supplement: Supplementary file 1. — Each horizontal entry represents the relevant genotype and allele transmission of the indicated diploids. The first column represents the diploid number, which matches the numbers shown in Figures 2–5. In columns 2–5, the two haploid parent strains (SZY#s) are indicated as are the alleles contributed by those parent strains at the experimental locus monitored for drive. Alleles derived from Sp are green whereas those from Sk are purple. For diploids 11–15, transmission of the wtf4 locus was followed using alleles of ura4, which is fortuitously closely linked to wtf4. Columns 9 and 10 indicate which phenotypes were followed at the drive loci and the number of progeny that displayed each phenotype. Some progeny inherited both alleles at a given drive locus and when the markers were codominant we could detect those disomes. The number of those disomes, which are likely heterozygous diploids or aneuploids, are shown in column 11 and their overall frequency is shown in column 12. If we did not have codominant markers, columns 11 and 12 are filled with ---. Column 13 is a measure of meiotic drive. It shows the fraction of the non-disomic progeny that inherited allele 1 (column 3). Column 14 shows the total number of progeny assayed for each diploid and column 15 is the p value calculated from a G-test comparing the allele transmission of allele 1 to a control. Diploid 13 served as the control for diploids 11, 12, 14 and 15. Diploid 16 served as the control for the rest of the diploids. Columns 6–8 are internal controls for each diploid. These controls represent an additional heterozygous locus unlinked to the meiotic drive locus that should undergo Mendelian allele transmission. The lys locus is lys4, the ade locus is ade6, and the ura locus is ura4. The final column indicates the number of independently generated diploids that were tested for each genotype. DOI: http://dx.doi.org/10.7554/eLife.26033.014 [file elife-26033-supp1.docx]

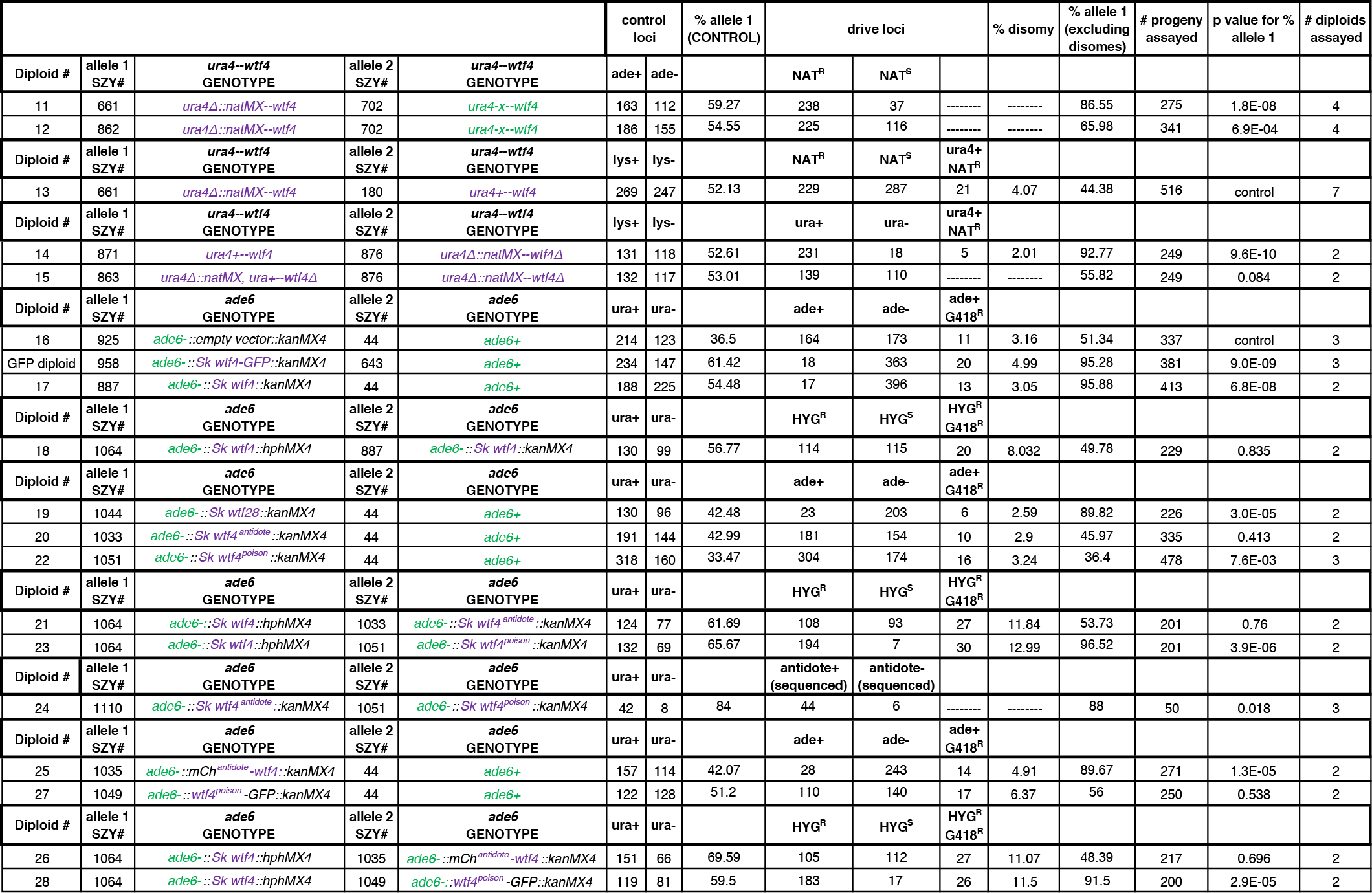


**Supplemental File 1: Raw data of allele transmission from Figures 2-5.**

Each horizontal entry represents the relevant genotype and allele transmission of the indicated diploids. The first column represents the diploid number, which matches the numbers shown in Figures 2-5. In columns 2-5, the two haploid parent strains (SZY#s) are indicated as are the alleles contributed by those parent strains at the experimental locus monitored for drive. Alleles derived from *Sp* are green whereas those from *Sk* are purple. For diploids 11-15, transmission of the *wtf4* locus was followed using alleles of *ura4*, which is fortuitously closely linked to *wtf4*. Columns 9 and 10 indicate which phenotypes were followed at the drive loci and the number of progeny that displayed each phenotype. Some progeny inherited both alleles at a given drive locus and when the markers were codominant we could detect those disomes. The number of those disomes, which are likely heterozygous diploids or aneuploids, are shown in column 11 and their overall frequency is shown in column 12. If we did not have codominant markers, columns 11 and 12 are filled with ---. Column 13 is a measure of meiotic drive. It shows the fraction of the non-disomic progeny that inherited allele 1 (column 3). Column 14 shows the total number of progeny assayed for each diploid and column 15 is the p value calculated from a G-test comparing the allele transmission of allele 1 to a control. Diploid 13 served as the control for diploids 11, 12, 14 and 15. Diploid 16 served as the control for the rest of the diploids. Columns 6-8 are internal controls for each diploid. These controls represent an additional heterozygous locus unlinked to the meiotic drive locus that should undergo Mendelian allele transmission. The *lys* locus is *lys4*, the *ade* locus is *ade6*, and the *ura* locus is *ura4*. The final column indicates the number of independently generated diploids that were tested for each genotype.
