# Supplementary material for: wtf genes are prolific dual poison-antidote meiotic drivers
Source: eLife. 2017 Jun 20;6:e26033. doi: 10.7554/eLife.26033 (PMC5478261; doi:10.7554/eLife.26033)
Supplement: Supplementary file 3. — DOI: http://dx.doi.org/10.7554/eLife.26033.016 [file elife-26033-supp3.docx]

**Supplemental File 3: Yeast strains**

| **Strain** | **Species** | **Genotype** |
| --- | --- | --- |
| SZY44 | Sp | *h-, lys4-95* |
| SZY120 | Sk | *h90, rec12Δ::ura4+, ura4Δ::kanMX4* |
| SZY80 | Sk | *h90, lys1Δ::kanMX4* |
| SZY192 | Sk | *h90, rec12Δ::ura4+, ura4Δ::kanMX4, his5Δ::natMX4MX4* |
| SZY196 | Sk | *h90, rec12Δ::ura4+, ura4Δ::kanMX4, lys1Δ::kanMX4* |
| SZY201 | Sp | *h-, lys1-37, rec12-171::ura4+, ura4-x* |
| SZY208 | Sk | *h90, rec12Δ::ura4+, ade6Δ::hphMX4, his5Δ::natMX4, ura4Δ::kanMX4* |
| SZY210 | Sk | *h90, ade6Δ::hphMX4, his5Δ::natMX4, ura4Δ::kanMX4* |
| SZY239 | hybrid with Sk karyotype | *h-, rec12Δ::ura4+, ade6Δ::hphMX4, ura4Δ::kanMX4,* |
| SZY247 | hybrid with Sk karyotype | *h?, rec12Δ::ura4+, lys1-37, his5Δ::natMX4, ura4-x,* |
| SZY320 | Sk | *h90, ura4Δ::natMX4* |
| SZY382 | hybrid with Sk karyotype | *h-, rec12∆::ura4+, lys1-37, ura4-x* |
| SZY547 | hybrid with Sk karyotype | *h-, rec12∆::ura4+, lys1-37, ura4-x, arg12∆::hphMX4* |
| SZY558 | hybrid with Sk karyotype | *h90, rec12Δ::ura4+, his5Δ::natMX4, ura4-x, arg12Δ::natMX4* |
| SZY562 | hybrid with Sk karyotype | *hybrid chr3, Sk chr1 and chr2. h90, rec12∆::ura4+, his5∆::natMX4, ura4-x, ade6∆::hphMX4* |
| SZY563 | hybrid with Sk karyotype | *hybrid chr3, Sk chr1 and chr2. h90, rec12∆::ura4+, his5∆::natMX4, ura4-x, ade6∆::hphMX4* |
| SZY564 | hybrid with Sk karyotype | *hybrid chr3, Sk chr1 and chr2. h90, rec12∆::ura4+, his5∆::natMX4, ura4-x, ade6∆::hphMX4* |
| SZY565 | hybrid with Sk karyotype | *hybrid chr3, Sk chr1 and chr2. h90, rec12∆::ura4+, his5∆::natMX4, ura4-x, ade6∆::hphMX4* |
| SZY566 | hybrid with Sk karyotype | *hybrid chr3, Sk chr1 and chr2. h90, rec12∆::ura4+, his5∆::natMX4, ura4-x, ade6∆::hphMX4* |
| SZY567 | hybrid with Sk karyotype | *hybrid chr3, Sk chr1 and chr2. h90, rec12∆::ura4+, his5∆::natMX4, ura4-x, ade6∆::hphMX4* |
| SZY574 | hybrid with Sk karyotype | *hybrid chr3, Sk chr1 and chr2. h90, rec12∆::ura4+, his5∆::natMX4, ura4-x, ade6∆::hphMX4* |
| SZY581 | hybrid with Sk karyotype | *hybrid chr3, Sk chr1 and chr2. h90, rec12∆::ura4+, his5∆::natMX4, ura4-x* |
| SZY582 | hybrid with Sk karyotype | *hybrid chr3, Sk chr1 and chr2. h90, rec12∆::ura4+, his5∆::natMX4, ura4-x* |
| SZY589 | hybrid with Sk karyotype | *hybrid chr3, Sk chr1 and chr2. h90, rec12∆::ura4+, his5∆::natMX4, ura4∆::kanMX4* |
| SZY591 | hybrid with Sk karyotype | *hybrid chr3, Sk chr1 and chr2. h90, rec12∆::ura4+, his5∆::natMX4, ura4∆::kanMX4* |
| SZY643 | Sp | *h90, leu1-32, ura4-D18* |
| SZY649 | hybrid with Sk karyotype | *h90, rec12Δ::ura4+, ura4-x, ade6Δ::hphMX4* |
| SZY659 | hybrid with Sk karyotype | *h90, rec12Δ::ura4+, ura4-x, ade6Δ::hphMX4, kanMX4@chr3 position 214,500bp* |
| SZY661 | Sk | *h90, ura4Δ::natMX4, leu1Δ::hphMX4* |
| SZY679 | hybrid with Sk karyotype | *ura4Δ::natMX4, kanMX4@chr3 position 214500bp, ade6Δ::hphMX4, rec12Δ::ura4+* |
| SZY684 | hybrid with Sk karyotype | *ura4Δ::natMX4, kanMX4@chr3 position 214500bp, ade6Δ::hphMX4, rec12Δ::ura4+* |
| SZY685 | hybrid with Sk karyotype | *ura4Δ::natMX4, kanMX4@chr3 position 214500bp, ade6Δ::hphMX4, rec12Δ::ura4+* |
| SZY686 | hybrid with Sk karyotype | *ura4Δ::natMX4, kanMX4@chr3 position 214500bp, ade6Δ::hphMX4, rec12Δ::ura4+* |
| SZY702 | hybrid with Sk karyotype | *h90, ura4-x, ade6Δ::hphMX4* |
| SZY862 | Sk | *h90, wtf4∆::kanMX4truncation(drugS), ura4∆::natMX4, leu1∆::hphMX4* |
| SZY863 | Sk | *h90, wtf4∆::kanMX4truncation(drugS), ura4∆::natMX4, leu1∆::hphMX4, ura4+* |
| SZY871 | Sk | *h90, leu1∆::hphMX4* |
| SZY873 | Sk | *h90, ura4∆::natMX4* |
| SZY876 | Sk | *h90, ura4∆::natMX4, lys1∆::kanMX4, wtf4∆::kanMX4(truncated drug S)* |
| SZY887 | Sp | *h90, leu1-32, ura4-D18, ade6-::Skwtf4::kanMX4::ade6-* |
| SZY925 | Sp | *h90, leu1-32, ura4-D18, ade6-::kanMX4::ade6-* |
| SZY960 | Sp | *h90, leu1-32, ura4-D18, ade6-::Skwtf4-GFP::kanMX4::ade6-* |
| SZY958 | Sp | *h-, lys4-95, ade6-::Skwtf4-GFP::kanMX4::ade6-* |
| SZY1030 | Sp | *h?, hht1-RFP::kanMX6, lys1-37* |
| SZY1033 | Sp | *h90, leu1-32, ura4-D18, ade6-::Skwtf4(357A>T, 358T>A, 359G>C)::kanMX4::ade6-* |
| SZY1035 | Sp | *h90, leu1-32, ura4-D18, ade6-::mCherry5Xglycine-Skwtf4::kanMX4::ade6-* |
| SZY1044 | Sp | *h90, leu1-32, ura4-D18, ade6-::Skwtf28::kanMX4::ade6-* |
| SZY1049 | Sp | *h90, leu1-32, ura4-D18, ade6-::Skwtf4(M1X, M12X)-GFP::kanMX4::ade6-* |
| SZY1051 | Sp | *h90, leu1-32, ura4-D18, ade6-::Skwtf4(M1X,M12X)::kanMX4::ade6-* |
| SZY1064 | Sp | *h-, lys4-95, ade6-::Skwtf4::hphMX4::ade6-* |
| SZY1072 | Sp | *h90, ura4-D18, leu1-32, hht1-RFP::kanMX4, ade6-::Skwtf4::hphMX4::ade6-* |
| SZY1095 | Sp | *h-, lys4-95, ade6-::Skwtf4-GFP::kanMX4::ade6-, his5Δ::ade6+* |
| SZY1110 | Sp | *h-, ade6-::Skwtf4(357A>T, 358T>A, 359G>C)::kanMX4::ade6-, lys4-95* |
| SZY1140 | Sp | *h90, hht1-CFP::hphMX4, his3-D1* |
| SZY1142 | Sp | *h90, ura4-D18, his5Δ::ade6+, lys1-37, ade6-::mCherry5Xglycine-Skwtf4::kanMX4::ade6-* |

Notes:

-The *ura4-X* allele (*e.g.* SZY382) listed in the table is either *ura4-D18* or *ura4-294*

-The *wtf4∆::kanMX4(truncated drug S)* allele is described in the methods. It does not confer

resistance to G418.

-The precise location of the *ura4^+^* allele in strain SZY863 is unknown. It is not at the

endogenous locus, although it is closely linked to the endogenous locus.

-The *ade6-::gene1::gene2::ade6-* allele naming structure is used in many strains. This indicates

that a plasmid derived from pSZB188 bearing two genes (*e.g. wtf4* and *kanMX4*) was integrated

into the *ade6^+^* locus. This recombination event generates two mutant copies of *ade6* flanking

the contents of the plasmid.

-The *Skwtf4(357A>T, 358T>A, 359G>C)* nomenclature denotes that the ATG sequence

beginning at position 357 of the *wtf4* gene has been changed to TAC. This is the ATG within

what is intron 1 of the antidote that used as the start codon for the poison transcript.

-The *Skwtf4(M1X,M12X)* nomenclature denotes that the first two methionines in the antidote

coding sequence have been mutated to stop codons (TAG).
