# Supplementary material for: wtf genes are prolific dual poison-antidote meiotic drivers
Source: eLife. 2017 Jun 20;6:e26033. doi: 10.7554/eLife.26033 (PMC5478261; doi:10.7554/eLife.26033)
Supplement: Supplementary file 4. — DOI: http://dx.doi.org/10.7554/eLife.26033.017 [file elife-26033-supp4.docx]

**Supplemental File 4: Plasmids**

| **plasmids** | **short description** | **reference** |
| --- | --- | --- |
| pFA6 | contains kanMX4 | 40 |
| pAG32 | contains hphMX4 | 39 |
| pSZB134 | contains cassette to target kanMX to position 214,491 in Sp chr 3 | this work |
| pSZB184 | pMZ283 expressing guide RNA to target wtf4 | this work |
| pMZ222 | contains Cas9 | 44 |
| pMZ283 | empty vector for expressing Cas9 guide RNA | 44 |
| pSZB136 | contains cassette to generate leu1∆::hphMX4 | this work |
| pKT127 | contains yEGFP | 30 |
| pSZB188 | derivative of pFA6a that integrates at ade6, yielding ade6- | this work |
| pSZB189 | pSZB188 with Sk wtf4 cloned into SacI site | this work |
| pSZB209 | pSZB188 with Sk wtf21 and wtf26 cloned into SacI site | this work |
| pSZB212 | pSZB188 with Sk wtf2 cloned into SacI site | this work |
| pSZB217 | pSZB188 with Sk wtf5 cloned into SacI site | this work |
| pSZB215 | pSZB188 with Sk wtf6 and wtf28 cloned into SacI site | this work |
| pSZB252 | pSZB188 with Sk wtf6 cloned into SacI site | this work |
| pSZB254 | pSZB188 with Sk wtf28 cloned into SacI site | this work |
| pSZB203 | pSZB188 with Sk wtf4-GFP cloned into SacI site | this work |
| pSZB204 | pSZB188 with Sk wtf4-GFP cloned into SacI site | this work |
| pSZB215 | pSZB188 with Sk wtf6 and wtf28 cloned into SacI site | this work |
| pSZB246 | pSZB188 with Sk wtf4antidote (ATG to TAC at nucleotide position 356) cloned into SacI site | this work |
| pSZB244 | pSZB188 with Sk wtf4 (ATG to TAG at nucleotide position 1) cloned into SacI site | this work |
| pSZB248 | pSZB188 with Sk mCherry-wtf4 cloned into SacI site | this work |
| pSZB252 | pSZB188 with Sk wtf6 cloned into SacI site | this work |
| pSZB254 | pSZB188 with Sk wtf28 cloned into SacI site | this work |
| pSZB257 | pSZB188 with Sk wtf4poison (ATG to TAG at nucleotide position 1 and ATG to TAG at position 34)-GFP cloned into SacI site | this work |
| pSZB258 | pSZB188 with Sk wtf4poison (ATG to TAG at nucleotide position 1 and ATG to TAG at position 34) cloned into SacI site | this work |
| pAG25 | contains natMX4 | 39 |
