# Supplementary material for: wtf genes are prolific dual poison-antidote meiotic drivers
Source: eLife. 2017 Jun 20;6:e26033. doi: 10.7554/eLife.26033 (PMC5478261; doi:10.7554/eLife.26033)
Supplement: Supplementary file 5. — DOI: http://dx.doi.org/10.7554/eLife.26033.018 [file elife-26033-supp5.docx]

**Supplemental File 5: Oligos**

| **Oligo** | **Sequence (5'-->3')** |
| --- | --- |
| 255 | TATTACATCAGTGCGCTATCAGTTTAAAAGGTTGGGCCTACTAACTTAACATATACTACACCTCAAGAAAAAGAAAGAACACATACGATTTAGGTGACAC |
| 256 | TTTGCACAGCAGAAATTTGATTATTGCTTGGCTCAAGTACATGGTGAGTATGACATTATTATTGAGAACGACCTGGCATAATACGACTCACTATAGGGAG |
| 380 | GTCGGATCCCATTCGTTATCGTTCCAAGTGTGCTGCCGTCG |
| 381 | GTCAGATCTCTGTTTTGGAAACTTTTTTATCCTCTAACGATGACGATAAATTTAC |
| 382 | GTCGAGCTCAATACAGGTAAATGGTCTAAATCAGTATGTAAGCC |
| 383 | GTCACTAGTGCTATGATTCCGGGAATTGATGTTTCTTCTGAC |
| 413 | CTCTGAAGACGCAGGTAGTAAAAAACCCG |
| 414 | GTGAGTATGTACCTTCAATACACCCTTGATG |
| 557 | CTGCGTAGCTGACATGTTATTGCGATAAC |
| 560 | GCATTGCTTGAAAGATTCTGCGATGTTGG |
| 565 | GGTTAGAGTAAATTACAGGAATATATAACGAACCC |
| 566 | GGGTTCGTTATATATTCCTGTAATTTACTCTAACC |
| 567 | CTACCTTGCCGAATATCGACTTCTCCAAC |
| 568 | GTTGGAGAAGTCGATATTCGGCAAGGTAG |
| 569 | CTGAACGAGGCAGTGGATTGCTTCTG |
| 570 | CAGAAGCAATCCACTGCCTCGTTCAG |
| 571 | GTTATGATGGAGAACCCGGAAATTAGAGGC |
| 572 | GTGTCACCTAAATCGTATGTGGGGAACAGAAATAAACAAGTCTAAAGTGCC |
| 573 | CTCCCTATAGTGAGTCGTATTAAACTGCGTAGCTGACATGACACTGAATTTC |
| 574 | CCAGGCAACATCCATTCTCATCAGATGAGG |
| 575 | ATTTCTGTTCCCCACATACGATTTAGGTGACAC |
| 576 | CAGCTACGCAGTTTAATACGACTCACTATAGGGAG |
| 577 | CATTTTCACAAATGGTTCGAGT |
| 578 | TCGAACCATTTGTGAAAATGTT |
| MESZ176 | TGGTTAAGCATGTGATCTTCATACGACGC |
| MESZ177 | AGAAATTCAGTGTCATGTCAGCTACGCAG |
| 588 | ATGAGCGAAAAACAGGTTGTAGGGATC |
| 589 | GGTACCTGACCTGAATTGTGAGGCCGAGG |
| 590 | CCATAGCAGCCAAAAGGGAGGGTTG |
| 591 | CACAATTCAGGTCAGGTACCCAACACCCAACTCTCGACTTCCAC |
| 595 | GGGTTGTAATGTTACCTATCACTAATATAGCTC |
| 597 | GCTTAATTATCATTTTTTCCATTTGTTTAATGGTTTAC |
| 598 | CGGGTAAGTAAAGAATCATTCATACAGTTGG |
| 599 | CCGCTAACACGCAGTTCGTCTTCC |
| 601 | CCCTTCTGAGACTACTAATATCAGTTCTTG |
| 602 | GGAATCGAGTCAGCAGTTGTTATCAACGGG |
| 603 | CCCGTTGATAACAACTGCTGACTCGATTCC |
| 604 | CTGAATATGGAGGCAATGTGCTCTCATC |
| 605 | ATGAAGAATAAAGATTATCCCTTGAGGTCGTCTATGG |
| 606 | TAAACCAGCACCGTCACCGACTTCGCTTTCAACTTCCATTTCCCCC |
| 607 | GGGGGAAATGGAAGTTGAAAGCGAAGTCGGTGACGGTGCTGGTTTA |
| 619 | AATATAGGAGCTCTGGTTAAGCATGTGATCTTCATACGACGC |
| 620 | AATATAGGAGCTCAGAAATTCAGTGTCATGTCAGCTACGCAG |
| 633 | AATATAGAGCTCAGAAATTCAGTGTCATGTCAGCTACGCAG |
| 634 | AATATAGAGCTCCGGGGACGAGGCAAGCTAAAC |
| 643 | AATATAGAGCTCAATGCTTCAAAATAATTTTGTAAATCATGTTATGCCG |
| 644 | AATATAGAGCTCTCTATCACAAAAAAGGTTGCAGCGGAGC |
| 647 | AATATAGAGCTCCGGCTACTGATAATTGCCTTGCACTCTTC |
| 648 | AATATAGAGCTCCGAATTGAGTTTGTAGGAAGAAACAAAGTTCC |
| 649 | AATATAGAGCTCGGCTCTTCGATGCAAAGTAAGGTAAGTAGTTG |
| 650 | AATATAGAGCTCCATCTCTAAACCCGTATTTGGTAGAAACGGC |
| 651 | AATATAGAGCTCCCGAAGTATCATATCAACGTAGTACACCATG |
| 652 | AATATAGAGCTCGGAGGCAAAGCCAAACGTTCTAGC |
| 686 | AATATAGGAGCTCTATAATAGATCACAAAGGAAAACTCGCCGCAG |
| 687 | AATATAGGAGCTCCTGCGTAGCTTACATGTTATTGCGATAACATTTCG |
| 701 | GTGTATATCATTCAATAATAGATTGTTTTTAAGAATAGAAGAATAAAGATTATCCC |
| 702 | GGGATAATCTTTATTCTTCTATTCTTAAAAACAATCTATTATTGAATGATATACAC |
| 732 | AATATAGAGCTCGCGTTATTAATGTAGTTGTCGCTACAGTTGG |
| 733 | AATATAGAGCTCCTTCTTATTCACCCCAACTTAGATTTCCTTATGCATC |
| 735 | CCAAATTTCAAAAGTTATTTATTTTATTATACCTTTCAGAAATTTGGAAATATATTAAAACTGTATCTGAAG |
| 736 | CTTCAGATACAGTTTTAATATATTTCCAAATTTCTGAAAGGTATAATAAAATAAATAACTTTTGAAATTTGG |
| 739 | GTACTCAATTCATCCtTAAGACGACCTCAAGGG |
| 740 | CCCTTGAGGTCGTCTTAGGATGAATTGAGTAC |
| 795 | AAATGGAATGCCCATCCTGATCTTAACTTG |
| 796 | TCCCTACAACCTGTTTTTCGCTCAT CGTGATGCAAAACTACTCTTTTCAATTAGA |
| 797 | AAAGTTGGTTGGAAAAATTATTCTGCATAG AGATGAATTGGATTATGTCAGGAAAAGAAC |
| 798 | GTGAACGATTAGGGACGAATTATCAACTGT |
| 799 | TCTAATTGAAAAGAGTAGTTTTGCATCACGATGAGCGAAAAACAGGTTGTAGGGA |
| 800 | GTTCTTTTCCTGACATAATCCAATTCATCTCTATGCAGAATAATTTTTCCAACCAACTTT |
| A01112 | TAACGCCGCCATCCAGTGTCG |
| 678 | GCCGAATATCGACTTCTCCAACGGG |
| 861 | GCCCTGTTAGACGAATTTATGCTCGTAATATGTC |
